# Analytical methods to assess the impacts of activity-based funding (ABF): a scoping review

**DOI:** 10.1186/s13561-021-00315-1

**Published:** 2021-05-18

**Authors:** Gintare Valentelyte, Conor Keegan, Jan Sorensen

**Affiliations:** 1 Structured Population and Health services Research Education (SPHeRE) Programme, Division of Population Health Sciences, Mercer Street Lower, Royal College of Surgeons in Ireland, Dublin, Ireland; 2 Healthcare Outcome Research Centre (HORC), Royal College of Surgeons in Ireland, Dublin, Ireland; 3 Economic and Social Research Institute (ESRI), Whitaker Square, Dublin, Ireland

**Keywords:** Activity-based funding, Hospital funding, Hospital performance, Outcomes, Analytical methodology

## Abstract

**Background:**

Activity-Based Funding (ABF) has been implemented across many countries as a means to incentivise efficient hospital care delivery and resource use. Previous reviews have assessed the impact of ABF implementation on a range of outcomes across health systems. However, no comprehensive review of the methods used to generate this evidence has been undertaken. The aim of this review is to identify and assess the analytical methods employed in research on ABF hospital performance outcomes.

**Methods:**

We conducted a scoping review in line with the Preferred Reporting Items for Systematic Reviews and Meta-Analyses extension for scoping reviews. Five academic databases and reference lists of included studies were used to identify studies assessing the impact of ABF on hospital performance outcomes. Peer-reviewed quantitative studies published between 2000 and 2019 considering ABF implementation outside the U.S. were included. Qualitative studies, policy discussions and commentaries were excluded. Abstracts and full text studies were double screened to ensure consistency. All analytical approaches and their relative strengths and weaknesses were charted and summarised.

**Results:**

We identified 19 studies that assessed hospital performance outcomes from introduction of ABF in England, Korea, Norway, Portugal, Israel, the Netherlands, Canada, Italy, Japan, Belgium, China, and Austria. Quasi-experimental methods were used across most reviewed studies. The most commonly used assessment methods were different forms of interrupted time series analyses. Few studies used difference-in-differences or similar methods to compare outcome changes over time relative to comparator groups. The main hospital performance outcome measures examined were case numbers, length of stay, mortality and readmission.

**Conclusions:**

Non-experimental study designs continue to be the most widely used method in the assessment of ABF impacts**.** Quasi-experimental approaches examining the impact of ABF implementation on outcomes relative to comparator groups not subject to the reform should be applied where possible to facilitate identification of effects. These approaches provide a more robust evidence-base for informing future financing reform and policy.

**Supplementary Information:**

The online version contains supplementary material available at 10.1186/s13561-021-00315-1.

## Background

### Activity-based funding

Activity Based Funding (ABF) has become the international model for funding hospital-based care and is referred to by many terms, such as case-mix funding, prospective payment system (PPS), payment by results (PbR) or fee-for-service (FFS) [1, 2]. Under ABF, hospitals are funded proportionately to their activity, creating a link between the number and type (case mix) of patients treated and the hospitals’ level of income [3]. Services provided to patients are reflected by an efficient price of providing those services and adjustments incorporated for different patient populations served. Prices are determined prospectively e.g. in terms of Diagnosis-Related Groups (DRGs), and reflect differences in hospital activity, based on types of diagnosis and procedures provided to patients [4]. DRGs provide transparent price differences, directly linking hospital services provision to hospital payments.

ABF is often implemented with the intention to provide hospitals with clearer incentives to achieve certain objectives. In particular, ABF is often meant to incentivise efficient hospital production, by allowing hospitals to keep any surplus for treatments with price above cost [3]. Efficiency may be achieved by treating more patients, given that payments are driven by the number of patient discharges [3, 5] or by providing greater quality of care, reflected by improved patient outcomes [5]. The advantages and disadvantages of ABF have been previously highlighted. The nature of ABF provides a natural ‘yardstick competition’ [6] where ‘money follows the patient’ [7], leading to increased levels of activity [8]. However, such increases in treatment level have been linked to patient selection issues such as ‘cream skimming’ i.e. where hospitals choose to treat more profitable patients, and ‘skimping’ less profitable patients within each DRG [9, 10]. Additionally, fixed payments per procedure have led to improvements in technical and cost efficiency [11, 12]. However, these incentives may impact on service quality. For example, ‘cream skimming’ impacts quality by over treating low-risk patients, and avoiding treatment of high-risk patients. Similarly, quicker patient discharge to ensure higher financial gain, has been shown to impact quality of care, with patients discharged ‘quicker and sicker’ [13].

### Previous reviews

Reviews to date, assessing the impacts of ABF, have primarily focused on whether ABF incentivises certain hospital performance outcomes. In many cases, attempts are to simply capture and identify the effects of the ABF policy, by reporting on a set of hospital outcomes. O’Reilly et al. (2012) [14] reported on the early experience with ABF implementation across five European countries. They found evidence that the introduction of ABF was associated with an increase in activity, a decline in length of stay (LOS) and/or a reduction in the rate of growth in hospital expenditure in most of the countries. However, they identified the short study periods and the lack of formal empirical evaluations as some of the key shortcomings in identifying any potential hospital impacts arising from ABF [14].

A recent scoping review of 135 articles summarised the empirical evidence on DRG-based hospital funding systems specific to Germany and Switzerland [15]. They identified case numbers, LOS and reimbursement/cost were the most frequently used outcome parameters to identify ABF effects. However, they concluded that overall empirical evidence was lacking due to limited empirical data used, with evidence primarily driven by personal opinions, assumptions and secondary analyses using limited data in the majority of the reviewed studies [15].

Similarly, in their review of 12 studies, Jakobsen (2010) [16] examined whether ABF was linked to improved efficiency across Scandinavian hospitals. He concluded that the research evidence painted a ‘*blurry picture’* of ABF’s effect on efficiency in the Scandinavian hospital sector, and questioned the reliability of the results [16]. He highlighted, the lack of examination and empirical modelling, reflecting a more accurate description of the real world, were the key factors to the inconclusive evidence [16].

Finally a systematic review and meta-analysis by Palmer et al. (2014) [2] focused on identifying the effects of ABF, compared to alternative funding systems, on specific outcomes: mortality, readmission, discharge destination, severity of illness and volume of care. They reviewed 65 studies (28 of which were from the U.S.) and reported overall varied outcome effects. They highlighted that ‘*Inferences regarding the impact of ABF are limited both by inevitable study design constraints (randomized trials of ABF are unlikely to be feasible) and by avoidable weaknesses in methodology of many studies* ’ [2].

Evidently, reviews to date provide mixed evidence of the hospital impacts arising from ABF implementation, with much of the evidence limited to a specific jurisdiction (Germany and Switzerland, Scandinavia and predominantly the U.S.). The blurry picture of ABF’s effect on hospital performance outcomes is mirrored across all the reviews, where the methodological limitations used to generate the evidence were described as empirically weak.

This scoping review seeks to provide a summary of the analytical methods used internationally for assessing outcomes related to the introduction of ABF mechanisms within the acute hospital sector. Specifically, this review seeks to provide a summary of the applied methods, in countries outside the U.S., where ABF implementation has been more recent, relative to the U.S. It is outside the scope of this review to report on the magnitude of the impacts of ABF, as reported across the reviewed studies. The scoping review methodology allows for inclusion of a wide variety of studies and presents a comprehensive overview of the analytical methods used to develop the current evidence.

### Aim

The aim of this scoping review is to identify and assess the analytical methods employed in research on ABF impacts on acute hospital outcomes. Specifically, reviews to date have focused on whether ABF incentivises certain hospital performance outcomes, however no comprehensive reviews have examined the analytical methodology used to generate this evidence. This is important as less robust methods create difficulties in identifying causal effects, particularly when experimental studies are not feasible, and researchers must rely on secondary data sources. Thus, a comprehensive summary of previously employed analytical methods and their relative trade-offs, will guide researchers towards adapting robust analytical approaches for health intervention and policy evaluation.

We address the following research questions:

- What analytical methods have been employed in the assessment of ABF impacts within the acute hospital setting?
- What are the trade-offs between the different methods employed?

### Non-experimental data methods

When examining the impact of an intervention or a change in policy, the main challenge is to determine whether the observed changes over time are attributable to the intervention or policy i.e. a causal effect. Ideally, this would be achieved through an experimental study design such as a randomised controlled trial (RCT) the gold standard approach, by comparing outcomes for a group subject to the intervention to a group not subject to the intervention. However, such experiments are rare in the field of health policy and financial economics, thus researchers need to identify alternative appropriate methods for exploiting non-experimental data.

Several methods have been proposed to be appropriate to use for non-experimental data. These consist of interrupted time series (ITS) [17], difference-in-differences (DiD) [18], synthetic control (SC) [19], matching [18] and instrumental variables (IV) [20] approaches (Table 1). In general these methods seek to identify the causal effect of an intervention (approximating experimental designs such as a RCT) and choice of method adopted is often determined by the characteristics of the observational data available for analysis.

**Table 1:** Summary of the key analytical methods used to assess health interventions and their relative trade-offs

Analytical method	Description	Advantages	Disadvantages	Trade-offs relative to other methods
Interrupted Time Series (ITS)	A before-after comparison in the level and trend of outcomes pre and post intervention [17, 21, 22]	Straightforward methodological approach without reliance on simplifying assumptions [17, 21, 22]	Influenced by simultaneous events occurring at the time of intervention [17, 21, 22]	No control group to compare intervention effects against a group exposed to the intervention which can bias estimated intervention effects [23]
Difference-in-differences (DiD)	A contrast of outcome changes pre and post intervention using a naturally occurring control group and treatment group subject to the intervention change [18, 24]	Using the intervention itself as a naturally occurring experiment, allows to difference out any exogenous effects from events occurring simultaneously [18, 24]	The parallel trends assumption is based on counter-factual intervention trends which cannot be tested [18, 24]	Use of a naturally occurring control group to compare intervention effects naturally isolates group differences from intervention effects. No statistical test to verify the parallel trends assumption can bias estimated effects [18, 24]
Synthetic Control (SC)	Comparison of treatment effects between a treatment group and a constructed control i.e. a synthetic control using weights similar to treatment outcomes pre-intervention [25, 26]	Can complement other analytical methods particularly when a naturally occurring control group cannot be established and/or when simplification assumptions do not hold e.g. the parallel trends assumption in DiD [25, 26]	Requirement of sufficient data pre and post intervention containing sufficient detail of control weights similar to the treatment group [19]	Can overcome parallel trends assumption required for DiD. Cannot test for similarity of controls used to construct the synthetic control which may bias estimated intervention effects. Heavy data requirement pre and post intervention [19, 25]
Matching	A comparison of outcomes between treatment and control groups pre and post intervention post matching groups with similar observable factors [18, 27]	Reduction of biases within groups is eliminated due to matching [18, 27]	Requirement of sufficient data pre and post intervention for matching similar observable characteristics between treatment and control groups. No statistical means to testing ‘similarity’ [27]	Heavy data requirement to match similar characteristics. Matching is limited to observable factors and does not account for non-observable factors. ‘Similarity’ determined using subjective judgment and cannot be statistically measured and can bias estimates [27].
Instrumental Variables (IV)	An observable variable i.e. the instrument is selected to randomise the estimation of treatment effects [18, 20, 28]	Introduction of randomness when estimating treatment effects to reflect similarity to a RCT [18]	Dependence on choosing the most appropriate instrument to satisfy the assumption of no relationship between the outcome and assuming outcome is affected only via intervention exposure [18, 29]	Imposed randomisation using an instrument useful for estimating intervention effects. Randomisation is imposed and not naturally occurring like with DiD and can bias estimated effects [18, 20, 28, 29]

The interrupted time series (ITS) analysis is one of the most commonly used quasi-experimental approaches for evaluating health policies and interventions. Using this approach, outcomes are measured at multiple time points before and after an intervention, allowing the change in level and trend of outcomes to be compared, and intervention effects estimated [17, 21]. The before-after comparison is within a single population, rather than a comparison with a control group, and can eliminate selection bias and limit confounding related to differences pre and post intervention [22]. This can be useful when estimating ABF impacts, as data used for analysis can often be limited to a single group of patients, a certain procedure or a group of hospitals. However, other events occurring around the time of the intervention can be a source of confounding and can lead to overestimation of the intervention effects [22]. For example, if the introduction of ABF at a point in time was accompanied by other changes to hospital policy or processes, then changes in measured outcomes could be wrongly attributed to the ABF introduction under the ITS design. Additionally, the ITS approach becomes less appropriate when other factors such as non-linear intervention trends, gradual implementation of the intervention over time, changes in population characteristics over time, and autocorrelation are present [17, 22]. If such factors are not accounted for, this can pose challenges in the estimation of ABF effects and can lead to an overestimation and consequently a misrepresentation of causal claims related to ABF.

An advantage of ITS is it being a simple method to estimate intervention effects. It does not rely on heavy data requirements, making full use of the longitudinal nature of the data analysed, and accounts for pre-intervention trends [17]. However, ITS analysis does not specify a control group against which effects on the group exposed to an intervention can be compared [18, 21]. A recent study tested the empirical strength of the ITS approach, by comparing the estimated ITS results to the results from a RCT [23]. They concluded that ITS produced large and completely misleading results, primarily driven by the lack of control group, and ITS model assumptions [23]**.** Thus, caution should be taken when considering ITS, as the estimates may not capture the effects of the intervention of interest.

Difference-in-differences (DiD) is another approach often used for evaluating intervention effects that addresses some of the shortcomings of the ITS design. Some of the limitations of the ITS approach can be overcome by specifying a control group not subject to the intervention of interest, and comparing outcomes pre and post intervention. Particularly, the DiD approach considers the intervention itself as a natural experiment, and finds a naturally occurring control group, similar to an experimental context [18, 24]. DiD identifies causal effects by contrasting outcome changes pre and post intervention, between treatment and control groups [18, 24]. For instance, when estimating ABF impacts, for the comparison to be meaningful, the selected control group must be similar to the treated group in the absence of the treatment along various dimensions. Often this is satisfied by the availability of sufficient data pre and post ABF implementation, allowing to make such comparisons feasible. However, it is important that an appropriate control group has been selected, and serves as a suitable control. Often using the DiD approach, a control group that appears to be natural relative to the treatment group is selected [18, 24].

The DiD estimators provide unbiased treatment effects under the assumption that the unobserved characteristics are fixed, and the average outcomes in each group would change in the same way in the absence of the intervention i.e. follow parallel trends [18, 19, 24, 25]. However, the parallel trends assumption applies to unobserved counterfactual post-intervention outcomes, which can never be statistically tested [18, 24]. This can pose challenges, as the presence of parallel trends pre-intervention does not guarantee these trends would continue in the absence of the intervention, and can lead to biased estimates of causal effects [18, 24]. Often the average outcome trends for the control and treatment groups in the pre-intervention period are examined visually [19] or using simple linear regression estimates [30]. Consequently, the key advantage of the DiD approach is that the estimates between the control and treatment groups naturally isolate any external shocks that may occur during the intervention period [18, 24]. Hence, robust estimates representing causal intervention effects are captured, as any possible biases related to permanent differences between the two groups, or biases that could influence the outcome trends are eliminated [24]. When evaluating ABF effects using the DiD approach, the captured effects are a result of ABF, as any impacts from other events that may have occurred simultaneously, are ‘differenced out’ [18]. The estimated treatment effects approximate a causal relationship between ABF only and the outcomes of interest.

The synthetic control (SC) method is becoming more widely applied in the evaluation of causal effects of health interventions. Like the DiD method, the SC method compares intervention effects between treatment and control groups. Using this approach, a synthetic control is constructed using the weighted average of the available control units [19, 25, 31]. The chosen weights are selected so that the outcomes and covariates between the treated unit and the synthetic control are similar to the outcomes in the pre-treatment period [19, 25, 26]. This approach becomes useful, particularly if the DiD parallel trends assumption cannot be established or may not hold [19, 26]. In contrast to the DiD parallel trends assumption, the SC approach assumes that the pre-intervention covariates have a linear relationship with outcomes post-intervention [19, 25]. However, the construction of the synthetic control unit relies on a set of controls that are similar to the treated unit, for which there is no consensus on how ‘similarity’ is measured [26]. Thus, the credibility of capturing robust intervention effects relies on constructing a good pre-intervention fit for the outcome of interest between the treated unit and the synthetic control [26]. Also, data availability with multiple suitable controls across treated and control units may not always be available, limiting the application of this method to certain types of data [26]. Although the commonly highlighted criticisms of the SC approach are related to the difficulty of interpreting the estimated results [26], and comparing these with other estimation methods [25], it has been recognised as a useful complementary approach to other quasi-experimental methods [18, 25, 26, 32].

Additionally, the matching method is applied to non-experimental data to capture intervention effects between control and treatment groups. Under this approach, observable factors of individuals with similar characteristics pre and post intervention are matched, and outcomes between these two groups compared [18]. A key advantage of this approach is the reduction of biases between both groups by making them similar using matching, which can improve causal inferences related to the treatment effects of an intervention [18, 27]. Similar to DiD and SC, any within group differences are eliminated and are isolated from the intervention effects [18]. However, matching is limited to the elimination of observable differences between treatment and control groups only, and any non-observable differences are not accounted for [27]. Additionally, deciding on the choice of the appropriate matching variables for both groups, is a commonly highlighted challenge [18, 27]. If the chosen covariates are not correctly defined, the intervention effects and consequently causal inferences will not be estimated correctly [18]. Like the SC method, ‘similarity’ between groups cannot be tested statistically, and often relies on varied researchers’ subjective judgment [18, 27]. In addition, there is a heavy data requirement of detailed information pre and post intervention for both the treatment and control groups [27]. For example, when evaluating ABF effects, this method could pose challenges, if data analysed are limited to a specific patient group, containing more detailed information post-intervention relative to the pre-intervention period. On the other hand, data that are too detailed, can make it more difficult to find a similar control group distinguishable from the treatment group [27]. Despite these shortcomings, the matching method is often combined with other methods e.g. DiD, and has been shown to significantly improve the quality and accuracy of the estimated intervention effects [25, 27].

Finally, instrumental variables (IV) method is another approach used in the evaluation of causal effects of an intervention based on non-experimental data. This method relies on the selection of an instrument, an observed variable which is assumed to be related to the intervention effect and is only related to the outcome through exposure to the intervention [18, 20, 28]. The instrument provides the required randomness for estimating the causal effects of an intervention, similar to a RCT, and allows for intervention effects to be separated, eliminating potential selection problems [18]. Similar to the matching approach, the randomisation imposed by the IV can also help to eliminate any group differences. However, under this method randomisation is imposed, rather than naturally occurring (e.g. under DiD), and the estimated intervention effects may be subject to bias [18]. Another key disadvantage of this approach is that it relies on choosing an appropriate instrument that will satisfy the assumption of no relationship between the instrument and the outcome [18]. For example, in their estimation of ABF impacts on elderly patients’ LOS, Yin et al. (2013) [33] used an instrument which introduced sufficient variation between individual patients and simultaneously consisted of time-invariant variables pre and post intervention. Similarly, in their estimation of hospital quality post ABF, Cooper et al. (2011) used an instrument for hospital competition, to impose variation in distance to patients’ closest hospitals [34]. However, the selection of such instruments are subject to the availability of data analysed, which should ideally consist of a large number of observations [29]. Additionally, a critique of the IV method is that the chosen instrument may affect the outcome through some pathway other than through the exposure of interest, which cannot be tested empirically [28]. Therefore, the estimated intervention effects may not be fully attributed to the intervention considering this potential relationship with the instrument.

Evidently, caution should be exercised, and the potential trade-offs considered, when choosing the appropriate estimation approach to capture the effects of a health policy such as ABF. Evidently, quasi-experimental analytical methods incorporating a control and treatment groups are preferred, as these are considered econometrically stronger methods to identify causal effects from an intervention.

## Methods

### Design

We used a systematic scoping review methodology in line with the Preferred Reporting Items for Systematic Reviews and Meta-Analyses extension for scoping reviews (PRISMA-ScR) as guidance throughout the reporting process [35]. We adopted the Arksey and O’Malley’s [36] methodological scoping review framework and followed guidance recommended by the Joanna Briggs Institute [37]. A protocol for this scoping review was registered on the Open Science Framework [38].

### Study selection

We constructed a search strategy with guidance from a research librarian, with necessary adaptations for the various search engines (Additional file 1: Appendix 1). We systematically searched five academic databases, including PubMed, Embase, EconLit, Web of Science, Health Business Elite for studies describing the impacts of ABF on hospital performance outcomes published between January 2000 and December 2019. All included articles were limited to publication in the English language. Additional articles deemed relevant were identified by hand searching reference lists of included final studies.

Studies were screened in two key stages; in the first stage all titles and abstracts and full texts were screened by the first author; in the second stage, a random portion of all abstracts and full texts were double screened by a second reviewer to ensure consistency. Any discrepancies between decisions were discussed by both reviewers, and a third researcher was available for consensus if necessary.

### Eligibility criteria

Studies were eligible for inclusion if they examined ABF impacts within the acute hospital setting, which use classification systems such as Diagnosis-Related Groups (DRGs) to derive hospital funding. We included studies published across countries other than the U.S. We limited our study inclusion to quantitative studies only.

We excluded studies describing the ABF process, studies focusing on ABF refinement or studies costing hospital services. Studies focusing on ABF impacts outside the acute hospital setting were not included. Additionally, studies that were based on examining ABF impacts in the U.S. were excluded, and studies that were not peer-reviewed research articles were also excluded. Conference abstracts and theoretical descriptive studies were also excluded.

The full inclusion and exclusion criteria with refinements from the study protocol can be found in Additional file 1: Appendix 2.

### Data-charting process

Study characteristics and data were agreed upon among the authors, and extracted in a Microsoft Excel data charting form by the first author. The data extraction included the following study characteristics: authors, year, country, analytical methodology, comparison group, and outcome measures.

## Results

### Study selection

**Fig. 1:**
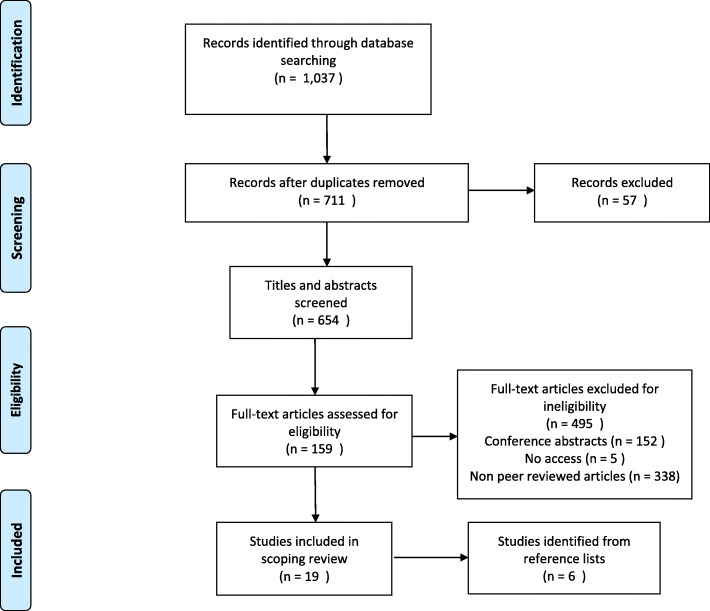
Preferred Reporting Items for Systematic Reviews and Meta-Analyses (PRISMA) Flowchart

A total of 1037 references were retrieved from the included databases (Fig. 1). After exclusion of duplicates, and other ineligible sources, 654 titles and abstracts were screened for eligibility, and 159 full texts screened after.

### Study characteristics

We identified a total of 19 studies which met our eligibility criteria (Additional file 1: Appendix 2). Of our included studies, four were conducted in England [30, 34, 39, 40], three each in Korea [41–43] and Norway [33, 44, 45], and one each in Portugal [46], Israel [47], the Netherlands [48], Canada [49], Italy [50], Japan [51], Belgium [52], China [53], and Austria [54]. Most studies were published after 2010 [30, 33, 34, 39, 41–43, 45, 48–51, 53], and six were published before 2010 [40, 44, 46, 47, 52, 54]. All included studies used quantitative methodology for data analysis, in their assessment of ABF impacts (see Table 2 and Additional file 1: Appendix 3).

**Table 2:** Analytical Methods used in order from early to most recent year of publication

Author(s) (Year)	Analytical methodology	Number of data points (frequency)		Comparison group	Level of analysis	Outcome measures
		Pre	Post			
Shmueli et al. (2002)	ITS: OLS Regression	2 (years)	1 (year)	No	Hospital	LOS; case numbers; mortality; readmission; real hospital income
Dismuke et al. (2002)	Count data models: Poisson & Negative Binomial regression	N/A	N/A	Yes: comparison of estimates between estimated models	Hospital	Mortality
Theurl (2007)	ITS: Regression models accounting for hospital, regional & DRG-specific effects	8 (years)	6 (years)	No	Hospital	LOS
Perelman et al. (2007)	ITS: Panel data regressions with hospital fixed effects	4 (years)	4 (years)	No	Hospital	LOS; medical cost; surgical cost
Farrar et al. (2009)	DiD: Accounting for DRG & hospital trust fixed effects	2/1 (years)	1 (year)	Yes: English & Scottish hospitals not implementing PbR	Hospital	LOS; case numbers; mortality; readmission
Martinussen et al. (2009)	ITS: OLS regression with hospital fixed effects	3 (years)	4 (years)	No	Procedure (DRG)	LOS
Cooper et al. (2011)	DiD: OLS regression & IV estimation with hospital fixed effects	4 (years)	3 (years)	Yes: DRGs not affected by PbR	Patient (DRG)	Mortality
Hamada et al. (2012)	DiD: Multilevel mixed-effect regression models	3 (years)	6 (years)	Yes: DRGs under FFS	Patient (DRG)	LOS; mortality; readmission; medical cost
Yin et al. (2013)	Log-linear regression & pseudo-panel IV estimation	N/A	N/A	Yes: effects compared across three ischemic heart diseases	Patient (DRG)	LOS
Kim H et al. (2015)	Descriptive methods: Descriptive statistics/ Student t-test/ Pearson chi-square test/ Fisher exact test	6 (months)	6 (months)	Yes: DRG effects before policy	Procedure (DRG)	LOS; mortality; readmission; outpatient visits; medical cost
Kim J et al. (2015)	Descriptive methods: Descriptive statistics/ Student’s t-test/ Pearson chi-squared test	8 (months)	8 (months)	Yes: DRG effects before policy	Procedure (DRG)	LOS; readmission; hospital cost; outpatient visits; adverse events; complication rates
Allen et al. (2016)	DiD: Differential-spline analyses to test for changes in the trend of an outcome	25 (months)	12 (months)	Yes: group of non-incentivised day surgeries	Procedure (DRG)	Case numbers
Januleviciute et al. (2016)	ITS: Fixed-effects regression models	2003 (base)		No	Procedure (DRG)	Case numbers
Sutherland et al. (2016)	ITS: Segmented GLM regression models accounting for Health Authority and hospital correlation over time	26 (months)	39 (months)	No	Hospital	LOS; case numbers; mortality; readmission
Verzulli et al. (2017)	DiD: Regression models accounting for differences between short & medium-run hospital effects	2 (years)	4 (years)	Yes: DRGs not affected by price change	Hospital	LOS; case numbers
Krabbe-Alkemade et al. (2017)	DiD: Log-linear regression models controlling for hospital market concentration & fixed effects	2 (years)	1 (year)	Yes: procedures under the budget reimbursement	Payment mechanism	Case number; DRG cost
Jung et al. (2018)	Descriptive methods: Descriptive statistics/ Pearson chi- squared test/ Student’s t-test	18 (months)	6 (months)	No	Procedure	LOS; Readmission;
Gaughan et al. (2019)	DiD & Synthetic Control approach	4 (years)	4.5 (years)	Yes: Non-incentivised same day discharge procedures	Procedure (DRG)	Case numbers
Zeng (2019)	Logistic regression analysis & Descriptive methods	N/A	N/A	Yes: comparing profit & loss making DRGs	Patient (DRG)	LOS; DRG cost

*ITS* Interrupted time series analysis, *DiD* Difference-in-differences analysis, *OLS* Ordinary-Least Squares, *IV* Instrumental Variable estimation, *GLM* Generalised-linear models, *LOS* Length of stay, *DRG* Diagnosis-Related Group, *PbR* Payment by Results – alternative term to ABF used in the UK, *FFS* Fee-for-service, *N/A* Information Not Available/Applicable

There was variation between the number of years of data that was analysed. Four studies analysed data over a 3 year period [39, 46–48], three studies analysed 7 years of data [33, 34, 44], two each analysed 8 years [51, 52], 6 years [50, 53], 4 years [40, 45], and 1 year [41, 42]. The remaining studies analysed data over 2 years [43], 9 years [30], 10 years [49] and 14 years [54] (Additional file 1: Appendix 3).

### Analytical method characteristics

Table 2 summarises the characteristics of the analytical methods across the 19 studies. The majority of studies employed a quasi-experimental methodological approach. Six studies used different variations of ITS analysis [44, 45, 47, 49, 52, 54], and seven studies used DiD methods [30, 34, 39, 40, 48, 50, 51]. The remaining studies applied a variation of methods: log-linear and pseudo-panel IV estimation [33], count data models using Poisson and Negative Binomial regressions [46], and four studies used descriptive methods [41–43, 53].

#### Interrupted time series

Among the six studies that employed ITS analysis, four compared the changes in outcome trends within a group of hospitals [47, 49, 52, 54], and two compared outcome trends across selected DRGs [44, 45]. Of these, four studies accounted for simultaneous policy effects, which may have impacted the estimated outcome trends allocated to ABF. Shmueli et al. (2002) examined short-term ABF effects across various hospital outcomes post-implementation, by comparing outcome changes from 1 year to the following year, to minimise capturing the effects of LOS initiatives which occurred simultaneously [47]. Theurl et al. (2007) assessed the impacts of ABF on the average LOS across a group of hospitals, by including province-specific determinants of LOS in their estimation, to isolate the effects of ABF [54]. Similarly, in their estimation of price differences based on hospital admission type, Januleviciute et al. (2016) included a linear time trend interaction with each DRG, to separate the price change effects on activity volume from other changes over time [45]. Sutherland et al. (2016) included an interaction to capture change over time concurrent to ABF, fixed hospital and health system effects, in their examination of changes across several hospital outcomes [49]. Perelman et al. (2007) examined the result of ABF implementation by analysing hospitals’ response in terms of in-patient LOS, medical and surgical expenditures [52]. To control for other factors that may have influenced changes across these outcomes, they included a linear time trend in their estimation model [52]. Finally, Martinussen et al. (2009), examined whether there was evidence of ‘cream skimming’ among surgical day-case DRGs, post ABF implementation, and accounted for potential concurrent policies, by including various time-specific and hospital-specific interaction variables [44].

#### Difference-in-differences/synthetic control

Varied control groups were selected among the studies that employed the DiD method. In their estimation of ABF effects across hospital trusts in England, Farrar et al. (2009) used a control group which combined non-hospital trusts in England and Scotland that did not implement ABF [40]. Gaughan et al. (2019) examined the impact of the introduction of same-day discharge price incentive across 32 incentivised conditions in England, against a control group of non-incentivised conditions with similar characteristics [30]. Additionally they applied the SC approach, against which the DiD estimates were compared. They used the same pool of control conditions as for DiD estimation. They constructed the synthetic control by assigning a non-negative weight to each control condition, which minimised the difference between the incentivised and SC conditions based on pre-policy same-day discharge rates [30]. They expressed these as the root mean squared prediction error, and average pre-policy patient characteristics [30]. Similarly in their analysis of the impact of introducing a Best Practice Tarriff (BPT) for a day case procedure, Allen et al. (2016) compared the effects against a control group of non-incentivised procedures recommended for day case treatment [39]. To test for changes in the outcome trends over time, they applied a spline regression and created knots in the pre-ABF period, to capture the anticipation and BPT effects [39]. In their analysis to determine whether greater exposure to market competition prompted hospitals to improve their performance in terms of quality, Cooper et al. (2011) estimated changes in acute myocardial infarction mortality trends, in the post ABF implementation period [34]. They compared their estimates between a control group of hospitals located in less competitive markets, relative to a treatment group of hospitals in more competitive markets [34]. Additionally, to account for endogeneity, they conducted an estimation using an IV to account for hospital competition [34]. In their examination of ABF effects for patients with acute myocardial infarction, Hamada et al. (2012) compared the differences between a control group which consisted of Fee-For-Service payments, to a treatment group of DRG-based payments [51]. Krabbe-Alkemade et al. (2017) examined the impact of ABF (market competition) across hospitals in the Netherlands, using DRGs that remained in the old budget-based payment system, as a control group [48]. Verzulli et al. (2017) examined the impacts of a price increase policy on public hospital performance, using a control group of DRGs not affected by the price change, and compared the estimated effects with a treatment group of DRGs affected by the price change [50].

Five of these studies reported on how the parallel trends assumption was examined [30, 34, 39, 48, 50]. Three studies examined the pre-intervention outcome trends visually [30, 39, 50]. In addition to visual examination, Gaughan et al. (2019) selected a single control condition, which minimised the difference in trends, by applying matching to pre-intervention outcome trends [30]. Allen et al. (2016) tested for significance between the incentivised procedure and the control procedures, by interacting with a linear trend measuring the months prior the policy implementation period [39]. If the coefficient measuring the difference between the procedures was zero, they assumed it followed parallel trends, and included it in their estimation model [39]. Krabbe-Alkemade et al. (2017) estimated their empirical model for separate years in the pre-ABF period, and estimates significant at 5%, were assumed to follow parallel trends [48]. In their estimation model, Cooper et al. (2011) accounted for pre-intervention parallel trends, by using two-part quarterly splines, interacted with measures of hospital competition [34].

#### Other analytical methods

The remaining studies employed varied methodological approaches. Using a log-linear regression and a pseudo-panel model with IV estimators, Yin et al. (2013) estimated the effect of ABF on hospital LOS for elderly patients with ischemic heart conditions [33]. After grouping individuals into pseudo-cohorts based on their characteristics, they included an IV which accounted for variation between individual patients and time-invariant variables and allowed for variation between some of the exogenous variables and individual-level random effects [33]. To capture differences, they compared ABF effects across three selected ischaemic heart diseases [33]. Dismuke et al. (2002) estimated a Poisson and a Negative Binomial count data models, in their assessment of ABF on hospital quality, as measured by in-patient mortality, for a selected DRG, accounting for the equidispersion assumption [46]. They contrasted their estimated effects between both models [46]. Zeng et al. (2019) evaluated the effects of ABF-based payment in a pilot hospital in Beijing, China, across 107 DRGs, to determine whether the payment reform resulted in a profit or loss for the hospital [53]. They estimated a logistic regression, along with descriptive methods of patient characteristics, to determine whether the hospital was making a profit or a loss, and identified which patient and DRG-specific characteristics had the greatest impact [53]. Kim H. et al. (2015) examined the effects of ABF payment at a single hospital in Korea, for appendectomies, across several clinical outcomes and medical costs [41]. Using descriptive methods they compared all continuous outcomes using a Students t-test, and categorical outcomes using a Pearson chi-square test [41]. They used the Fisher exact test for linearly associated outcomes, outcomes with less than 5 observations and outcomes that consisted of more than two categories [41]. Similarly, Kim J. et al. (2015) assessed the impact of ABF on the use of medical resources and the rate of adverse events for patients undergoing laparoscopic appendectomy [42]. They used descriptive analysis to compare differences between various outcomes, using Students t-test and Pearson chi-square test for continuous and categorical outcomes, respectively [42]. Jung et al. (2018), examined the effects of ABF on the quality of several obstetrics and gynaecology procedures, using descriptive methods consisting of chi-squared tests and Students t-tests to compare differences across outcomes [43].

The outcome measures of hospital performance varied across the reviewed studies (Table 2). The most frequently used outcome measures were case numbers [30, 39, 40, 45, 47–50], LOS [33, 40–44, 47, 49–54], mortality [34, 40, 41, 46, 47, 49, 51] and readmission [40–43, 47, 49, 51]. (See Additional file 1: Appendix 4 for a more detailed summary of the analytical methods used in each study.)

## Discussion

While variation in the methodological strength of studies has previously been identified [2], reviews to date have focused on whether ABF incentivises certain hospital performance outcomes, rather than the analytical methods used to generate this evidence. The quality of the analytical methods is important, as better methods generate more robust evidence on which to base hospital financing and policy decisions. To our knowledge, this is the first review providing a detailed summary of the statistical methods used to generate evidence on the relationship between ABF implementation and hospital performance outcomes.

Overall, we identified a variation in the types of analytical methods applied over the study review period. It appears that one of the conventional analytical methods in the estimation of ABF effects, is the ITS approach [44, 45, 47, 49, 52, 54]. As described above, the ITS method does not rely on heavy data requirements, and would be considered the simplest approach used to estimate intervention effects relative to the other approaches [17]. However, this creates problems as using the ITS approach any shifts in the level or trend at the time of the intervention’s introduction, are fully attributable to the intervention itself [17]. To overcome potential influences on ABF estimates, due to external factors or concurrent policies, the reviewed studies included linear time trends and interactions [44, 45, 49, 52], outcome specific controls [54], or compared outcome effects for each year [47]. Given the varied approaches to eliminating potential biases influencing ITS estimates, caution should be taken, as these may not fully eliminate the external influences on the estimated ABF effects. Additionally, by not having a control group, the ITS method is at risk of producing misleading estimation results [23]. The key advantage of having a control group is the possibility to difference out any unmeasured group or time-invariant confounders from the intervention itself [18]. For example, the implementation of ABF may be accompanied by a change in hospital discharge policies, aimed at reducing LOS. In this case, the ITS approach may attribute the reduction in LOS, and capture this as the impact of ABF entirely, although this effect is that of the hospital policy. However, by having a control group, (e.g. patients subject to the LOS policy but not subject to ABF implementation) the ABF effect would be ‘differenced out’ from the LOS reduction policy to capture the impacts related to ABF only. It is evident, that the ITS approach is empirically weaker, which has previously been addressed [23], and caution must be taken when considering employing this method in policy and health intervention evaluation.

Although non-experimental data methods such as DiD have been identified as more suitable and robust in nature [18], only seven studies in this review employed this approach [30, 34, 39, 40, 48, 50, 51]. To ensure non biased estimated intervention effects, the DiD approach relies on sufficient examination of parallel trends and the selection the most appropriate control group [18]. Although the parallel trends assumption cannot be tested, five studies in this review described their varied approaches used in examining parallel trends [30, 34, 39, 48, 50]. This can pose challenges, as the different examinations do not suggest the correct approach was undertaken. Consequently, this may impact the estimated intervention effects across the chosen hospital performance outcomes.

Similarly, the chosen control groups among these studies varied. For example, Farrar et al. (2009) used a control group of hospital trusts from a different country (Scotland), which was deemed to be comparable to the treatment group of hospital trusts in the UK [40]. The authors assumed that the hospital system in the UK could be directly comparable to that in Scotland. However, the choice of the control group in this case is questionable, as well as the estimated ABF effects. The DiD approach establishes a control group that is more naturally occurring, similar to an experimental context [18]. Therefore, estimation of ABF effects in the UK, using a control group of hospitals from a different country would appear less appropriate in this case. A control group within the UK hospital system would seem more appropriate, and would be prone to less biased estimated effects, specific to the UK context. In contrast, several studies used a control group of procedures (DRGs) [30, 39, 48, 50, 51] or hospitals [34] not subject to the ABF change. Evidently, these control groups appear to be more naturally occurring and more appropriate for estimating ABF impacts. However, it is important that the selected procedures or hospitals in the control group, can be comparable with estimates of the treatment group.

Consequently, it may not always be possible to identify a naturally occurring control group. In such instances, the SC method provides a solution, allowing the control group i.e. the synthetic control to be constructed from a set of weights similar to the treatment group [18]. One study in our review applied the SC method in their estimation [30]. Their synthetic control group consisted of non-negative weights across several control conditions with the smallest difference between the incentivised conditions, in the pre-ABF period [30]. To ensure the appropriate synthetic control is constructed, it has been highlighted that the chosen weights in the pre-intervention period, must be similar to the covariates and outcomes over time, to those of the treatment group [25]. However, the application of the SC method in the evaluation of health policies and interventions is somewhat limited [25]. Thus, it is difficult to confirm whether the most appropriate synthetic control group has been constructed, particularly in the assessment of ABF impacts. Additionally, given the heavy data requirements for using this method, researchers may be limited in their construction of the most appropriate control group.

In addition to estimation of ABF effects between treatment and control groups, other studies combined their DiD approach with other methods to strengthen the robustness of their findings. Gaughan et al. (2019) compared their DiD estimates with estimates from the SC method [30]. Allen et al. (2016) [39] combined their DiD analysis with differential spline analyses to capture the true effects of the implementation of best practice tariffs for day case surgery. Similarly, in their estimation of ABF impacts on hospital quality, Cooper et al. (2011) [34] applied the IV approach to check the robustness of their DiD estimates. Their instrument accounted for hospital competition by incorporating variation in distance to patients’ nearest hospitals [34]. Evidently, results from these studies would appear more robust in nature than using DiD estimation alone, improving the causal inferences related to ABF effects.

It can be argued that the better designed DiD studies tend to have more pre and post-intervention time points in their estimation of ABF impacts. Among the reviewed studies, several conducted the DiD estimation using data over much longer time periods relative to other studies (Additional file 1: Appendix 3). Inclusion of more data points pre and post-intervention would allow to account for more characteristics which could impact the intervention effects, and could improve the robustness of the estimates. Additionally, it is difficult to draw inferences from analysis conducted over a short time frame, as the captured effects are often short-term in nature, and may not be truly reflective of the policy under consideration. Policy impacts may have already been anticipated prior to implementation, thus the estimates may not truly be reflective of post implementation effects. For example, ABF is often implemented with longer term objectives under consideration, thus inferences drawn from analysis conducted over a short period, both pre and post implementation, may not capture true policy effects. However, it is important to note that certain contexts may have limited access to more complete types and nature of data, thus limiting the timeframe of analysis.

Similarly, we identified studies that used simpler analytical methods, such as descriptive approaches [41–43, 46, 53]. Drawing conclusions from these studies must be done with great caution, as comparisons often do not reflect causal effects. Evidently, these methods were limited by data type and quality available for analysis, as reflected by the varied number of countries across which the reviewed studies were conducted.

Finally, it is evident that the variation of the measured hospital performance outcomes is directly related to the choice of analytical methods employed in assessing ABF impacts. All of the studies that employed the ITS method, the main outcome measure estimated was LOS [44, 47, 49, 52, 54]. Similarly, the studies that used the DiD approach, the main outcome measure consisted of case numbers [30, 39, 40, 48]. Evidently, the chosen analytical approach affects the type of outcome measures suitable for estimation. Also, the nature of the non-experimental data at researchers’ disposal plays a factor in the types of outcomes that can be estimated.

Future research in the ABF context should consider adopting more sophisticated analytical methods, to ensure the estimated intervention effects approximate experimental designs such as a RCT. Control-treatment methods such as DiD are more robust, given their nature allowing to compare effects across treatment and comparator groups [24]. Additionally, more advanced analytical methods could be used in addition to or instead of DiD, which are rarely applied to ABF, as addressed in this review. Methods such as matching, and SC could be applied, which also rely on estimating and comparing policy effects between control and treatment groups [18, 25].

### Limitations

This study has a number of limitations. First, we potentially could have missed studies by not searching grey literature and other databases but we do not see these limitations biasing the representativeness of studies measuring the impacts of ABF. Second, we did not systematically evaluate the quality or risk of bias of the studies, as the primary focus was on the quality of the analytical methods applied, and not the concluding results of the studies. Finally, by limiting our review to studies conducted outside of the U.S., we have reduced the level of evidence on ABF research. However, this was due to our interest to focus on the methods used in countries where ABF has been introduced more recently, relative to the U.S. Despite some limitations, in this review we have identified the types of analytical methods used in ABF research. We provide a summary of these methods which can be used by policy and decision makers to better inform future policy. Finally, we have addresseed the relative methodological trade-offs to help inform and guide future research focusing on the evaluation of health and policy interventions.

## Conclusions

This scoping review identified and summarised the analytical methods employed in research on ABF hospital performance outcomes. Non-experimental study designs continue to be the widely used method in the assessment of hospital impacts post ABF implementation. The findings of this study accentuate the need for more sophisticated quasi-experimental approaches to be used. Such approaches will provide more robust evidence for informing future financing reform and policy. We hope that the comprehensive summary of previously employed analytical methods and their relative trade-offs, will help guide and inform researchers and relevant policy stakeholders towards adapting robust analytical approaches for health and policy evaluations.

## Supplementary Information

**Additional file 1: Appendix 1.** Sample search strategy. **Appendix 2.** Study inclusion and exclusion criteria. **Appendix 3.** Summary table of included study characteristics. **Appendix 4**. Analytical Methodology - Summary by study.

## Data Availability

Not applicable.

## Abbreviations

ABF: Activity-Based Funding
DRG: Diagnosis-Related Group
RCT: Randomised Controlled Trial
IV: Instrumental Variables
DiD: Difference-in differences
SC: Synthetic Control
PRISMA-ScR: Preferred Reporting Items for Systematic Reviews and Meta-Analyses extension for scoping reviews
OLS: Ordinary Least Squares
ITS: Interrupted-time series
GLM: Generalised Linear Model
LOS: Length of Stay
CT: Computerised tomography
ICU: Intensive Care Unit
GP: General Practitioner
FFS: Fee-for-service
BPT: Best Practice Tariff
PbR: Payment by Results
PPS: Prospective Payment System

## References

[1] Baxter PE, Hewko SJ, Pfaff KA, Cleghorn L, Cunningham BJ, Elston D, Cummings GG (2015). Leaders' experiences and perceptions implementing activity-based funding and pay-for-performance hospital funding models: a systematic review. Health Policy.

[2] Palmer KS, Agoritsas T, Martin D, Scott T, Mulla SM, Miller AP, Agarwal A, Bresnahan A, Hazzan AA, Jeffery RA, Merglen A, Negm A, Siemieniuk RA, Bhatnagar N, Dhalla IA, Lavis JN, You JJ, Duckett SJ, Guyatt GH (2014). Activity-based funding of hospitals and its impact on mortality, readmission, discharge destination, severity of illness, and volume of care: a systematic review and meta-analysis. PLoS One.

[3] Street A, Vitikainen K, Bjorvatn A, Hvenegaard A (2007). Introducing activity-based financing: a review of experience in Australia, Denmark, Norway and Sweden. Working Papers 030cherp, Centre for Health Economics, University of York.

[4] Street A, Maynard A (2007). Activity based financing in England: the need for continual refinement of payment by results. Health Econ Policy Law.

[5] Brick A, Nolan A, O'Reilly J, Smith S (2010). Resource Allocation, Financing and Sustainability in Health Care. Evidence for the Expert Group on Resource Allocation and Financing in the Health Sector.

[6] Shleifer A (1985). A theory of yardstick competition. RAND J Econ.

[7] McElroy B, Murphy A (2014). An economic analysis of money follows the patient. Ir J Med Sci.

[8] Kjerstad E (2003). Prospective funding of general hospitals in Norway—incentives for higher production?. Int J Health Care Finance Econ.

[9] Ellis RP (1998). Creaming, skimping and dumping: provider competition on the intensive and extensive margins. J Health Econ.

[10] Cheng TC, Haisken-DeNew JP, Yong J (2015). Cream skimming and hospital transfers in a mixed public-private system. Soc Sci Med.

[11] Biørn E, Hagen TP, Iversen T, Magnussen J (2003). The effect of activity-based financing on hospital efficiency: a panel data analysis of DEA efficiency scores 1992-2000. Health Care Manag Sci.

[12] Biørn E, Hagen TP, Iversen T, Magnussen J (2010). How different are hospitals' responses to a financial reform? The impact on efficiency of activity-based financing. Health Care Manag Sci.

[13] Qian X, Russell LB, Valiyeva E, Miller JE (2011). Quicker and sicker' under Medicare's prospective payment system for hospitals: new evidence on an old issue from a national longitudinal survey. Bull Econ Res.

[14] O'Reilly J, Busse R, Hakkinen U, Or Z, Street A, Wiley M (2012). Paying for hospital care: the experience with implementing activity-based funding in five European countries. Health Econ Policy Law.

[15] Koné I, Zimmermann BM, Nordström K, Elger BS, Wangmo T (2019). A scoping review of empirical evidence on the impacts of the DRG introduction in Germany and Switzerland. Int J Health Plann Manag.

[16] Jakobsen MLF (2010). The effects of new public management: activity-based reimbursement and efficiency in the Scandinavian hospital sectors. Scand Polit Stud.

[17] Kontopantelis E, Doran T, Springate DA, Buchan I, Reeves D (2015). Regression based quasi-experimental approach when randomisation is not an option: interrupted time series analysis. Bmj..

[18] Blundell R, Costa DM (2000). Evaluation methods for non-experimental data. Fisc Stud.

[19] Kreif N, Grieve R, Hangartner D, Turner AJ, Nikolova S, Sutton M (2016). Examination of the synthetic control method for evaluating health policies with multiple treated units. Health Econ.

[20] Zhang Z, Uddin MJ, Cheng J, Huang T (2018). Instrumental variable analysis in the presence of unmeasured confounding. Ann Transl Med.

[21] Hudson J, Fielding S, Ramsay CR (2019). Methodology and reporting characteristics of studies using interrupted time series design in healthcare. BMC Med Res Methodol.

[22] Bernal JL, Cummins S, Gasparrini A (2017). Interrupted time series regression for the evaluation of public health interventions: a tutorial. Int J Epidemiol.

[23] Baicker KST (2019). Testing the Validity of the Single Interrupted Time Series Design. CID Working Papers 364, Center for International Development at Harvard University.

[24] Angrist JDP, Jorn-Steffen (2009). Parallel Worlds: Fixed Effects, Differences-in-differences, and Panel Data. Mostly Harmless Econometrics: An Empiricist's Companion: Princeton University Press.

[25] O'Neill S, Kreif N, Grieve R, Sutton M, Sekhon JS (2016). Estimating causal effects: considering three alternatives to difference-in-differences estimation. Health Serv Outcome Res Methodol.

[26] Bouttell J, Craig P, Lewsey J, Robinson M, Popham F (2018). Synthetic control methodology as a tool for evaluating population-level health interventions. J Epidemiol Community Health.

[27] Stuart EA (2010). Matching methods for causal inference: a review and a look forward. Stat Sci.

[28] Uddin MJ, Groenwold RH, Td B, Belitser SV, Roes KC, Klungel OH (2015). Instrumental Variable Analysis in Epidemiologic Studies: An Overview of the Estimation Methods. Pharmaceutica Analytica Acta.

[29] Angrist JD, Krueger AB (2001). Instrumental variables and the search for identification: from supply and demand to natural experiments. J Econ Perspect.

[30] Gaughan J, Gutacker N, Grašič K, Kreif N, Siciliani L, Street A (2019). Paying for efficiency: Incentivising same-day discharges in the English NHS. J Health Econ.

[31] Abadie A, Diamond A, Hainmueller J (2010). Synthetic control methods for comparative case studies: estimating the effect of California’s tobacco control program. J Am Stat Assoc.

[32] Lopez Bernal J, Cummins S, Gasparrini A (2019). Difference in difference, controlled interrupted time series and synthetic controls. Int J Epidemiol.

[33] Yin J, Lurås H, Hagen TP, Dahl FA (2013). The effect of activity-based financing on hospital length of stay for elderly patients suffering from heart diseases in Norway. BMC Health Serv Res.

[34] Cooper Z, Gibbons S, Jones S, McGuire A (2011). Does hospital competition save lives? Evidence from the English NHS patient choice reforms*. Econ J.

[35] Tricco AC, Lillie E, Zarin W, O'Brien KK, Colquhoun H, Levac D, Moher D, Peters MDJ, Horsley T, Weeks L, Hempel S, Akl EA, Chang C, McGowan J, Stewart L, Hartling L, Aldcroft A, Wilson MG, Garritty C, Lewin S, Godfrey CM, Macdonald MT, Langlois EV, Soares-Weiser K, Moriarty J, Clifford T, Tunçalp Ö, Straus SE (2018). PRISMA extension for scoping reviews (PRISMA-ScR): checklist and explanation. Ann Intern Med.

[36] Arksey H, O'Malley L (2005). Scoping studies: towards a methodological framework. Int J Soc Res Methodol.

[37] Peters MDJ GC, McInerney P, Munn Z, Tricco AC, Khalil, H. Chapter 11: Scoping Reviews (2020 version). In: Aromataris E, Munn Z (Editors). JBI; 2020 [Available from: https://synthesismanual.jbi.global.

[38] Valentelyte G. The impact of Activity-Based Funding (ABF) on hospital performance: a scoping review protocol 2019 [Available from: doi: 10.17605/OSF.IO/ZU5FK.

[39] Allen T, Fichera E, Sutton M (2016). Can payers use prices to improve quality? Evidence from English hospitals. Health Econ.

[40] Farrar S, Yi D, Sutton M, Chalkley M, Sussex J, Scott A (2009). Has payment by results affected the way that English hospitals provide care? Difference-in-differences analysis. BMJ (Online).

[41] Kim H, Jung IM, Yun KW, Heo SC, Ahn YJ, Hwang K-T, Lee HW, Koo DH, Ko E, Ahn HS, Shin R, Chung JK (2015). Early outcome of the Korean diagnosis-related groups payment system for appendectomy. Ann Surg Treat Res.

[42] Kim JW, Shin DW, Chae JJ, Kim JY, Park SG. Impact of the new payment system on laparoscopic appendectomy in Korea. J Surg Res. 2015. 10.1016/j.jss.2015.04.070 26025628

[43] Jung YW, Pak H, Lee I, Kim EH (2018). The effect of diagnosis-related group payment system on quality of Care in the Field of obstetrics and gynecology among Korean tertiary hospitals. Yonsei Med J.

[44] Martinussen PE, Hagen TP (2009). Reimbursement systems, organisational forms and patient selection: evidence from day surgery in Norway. Health Econ Policy Law.

[45] Januleviciute J, Askildsen JE, Kaarboe O, Siciliani L, Sutton M (2016). How do hospitals respond to Price changes? Evidence from Norway. Health Econ (United Kingdom).

[46] Dismuke CE, Guimaraes P (2002). Has the caveat of case-mix based payment influenced the quality of inpatient hospital care in Portugal?. Appl Econ.

[47] Shmueli A, Intrator O, Israeli A (2002). The effects of introducing prospective payments to general hospitals on length of stay, quality of care, and hospitals’ income: the early experience of Israel. Soc Sci Med.

[48] Krabbe-Alkemade YJFM, Groot TLCM, Lindeboom M (2017). Competition in the Dutch hospital sector: an analysis of health care volume and cost. Eur J Health Econ.

[49] Sutherland JM, Liu G, Crump RT, Law M (2016). Paying for volume: British Columbia's experiment with funding hospitals based on activity. Health Policy.

[50] Verzulli R, Fiorentini G, Lippi Bruni M, Ugolini C (2017). Price changes in regulated healthcare markets: do public hospitals respond and how?. Health Econ.

[51] Hamada H, Sekimoto M, Imanaka Y (2012). Effects of the per diem prospective payment system with DRG-like grouping system (DPC/PDPS) on resource usage and healthcare quality in Japan. Health Policy.

[52] Perelman J, Closon MC (2007). Hospital response to prospective financing of in-patient days: the Belgian case. Health Policy.

[53] Zeng J-Q (2019). The pilot results of 47 148 cases of BJ-DRGs-based payment in China. Int J Health Plann Manag.

[54] Theurl E, Winner H (2007). The impact of hospital financing on the length of stay: evidence from Austria. Health Policy.

