# Supplementary material for: Analytical methods to assess the impacts of activity-based funding (ABF): a scoping review
Source: Health Econ Rev. 2021 May 18;11:17. doi: 10.1186/s13561-021-00315-1 (PMC8132407; doi:10.1186/s13561-021-00315-1)
Supplement: Supplementary file 1 — Additional file 1: Appendix 1. Sample search strategy. Appendix 2. Study inclusion and exclusion criteria. Appendix 3. Summary table of included study characteristics. Appendix 4. Analytical Methodology - Summary by study. [file 13561_2021_315_MOESM1_ESM.docx]

**Appendix 1: Sample search strategy**

|  | Search string | Boolean operators |
| --- | --- | --- |
| 1 | “activity based funding” OR “activity based financing” OR “activity based reimbursement” OR “prospective payment system” OR “prospective funding system” OR “prospective financing system” OR “payment by results” OR “case-mix funding” OR “volume based funding” OR “volume based financing” OR “volume based reimbursement” OR “service based funding” OR “service based financing” OR “service based reimbursement” OR “patient based funding” |  |
|  |  | **AND** |
| 2 | “hospital” OR “hospital service” OR “hospital department” OR “hospital ward” OR “emergency department” OR “emergency ward” OR “emergency room” OR “medical clinic” OR “medical institution” |  |
|  |  | **AND** |
| 3 | Publication year: 2000 – 2019  English language |  |

**Appendix 2: Study inclusion and exclusion criteria**

*Inclusion criteria:*

- Studies conducted in countries that use classification systems as means of deriving hospital funding e.g. DRGs
- Studies focusing on acute hospital activities – surgical and medical admissions e.g. inpatients, day cases - (emergency and elective type) – [ excludes specific care of elderly, psychiatry]
- Studies reporting various hospital performance outcomes relating to ABF
- Studies published between years 2000 and 2019 (recent review by Palmer et al. on ABF included many studies published prior to 2000) – due to research staff limitations and time limitations
- Studies published in English

*Exclusion criteria:*

- U.S. studies: interested in studies that have implemented ABF more recently relative to the U.S. i.e. ‘the gold standard’ where ABF was implemented in 1983 and has advanced ahead of most countries
- Studies focusing on ABF effects outside the hospital setting i.e. primary care
- Studies describing ABF funding mechanism and/or the ABF price setting process
- Studies costing hospital services and not reporting on ABF impacts
- Studies addressing ABF refinement as result of resource group development e.g. improved DRG clinical coding systems
- Studies focusing on other volume-based funding mechanisms/ variations of ABF e.g. fee-for-service
- Non peer-reviewed research articles e.g. Reports, Notes, Policy documents etc.
- Theoretical studies
- Qualitative studies
- Literature reviews
- Conference abstracts
- Studies specific to Emergency Department (ED) services – ED care is not acute and is representative of follow-up/emergency care post hospital discharge
- Studies specific to Intensive Care Unit (ICU) services - ICU represents a single component of full care trajectory, and often consists of variations of costing studies
- Studies specific to maternity care and services – given these services are often varied and are provided across different settings e.g. at maternity specific hospitals, outside the acute hospital setting, it was decided these should not be included in the review

Further clarifications regarding the inclusion and exclusion criteria:

- **Studies conducted in the USA were excluded:** The USA was the first place to implement the Prospective Payment System (PPS)/(ABF) in 1983, and has most experience and progressed most in using this funding system than most countries that have adapted this in the last 20 years (also a reason for selecting studies published from 2000 to 2019). Additionally, the USA healthcare system is primarily insurance driven, and considered outside the scope of this review, where the focus is on ABF impacts across non insurance driven healthcare systems i.e. consisting of public healthcare and/or a combination of private insurance funding – which is the case across most countries outside the USA

**Appendix 3: Summary table of included study characteristics**

| **Author(s)** | **Year** | **Study country** | **Years of data (no. of years)** |
| --- | --- | --- | --- |
| Allen et al. | 2016 | England | Dec 2007 – March 2011  (3y 3m) |
| Kim H et al. | 2015 | Korea | July 2012 – June 2013 (1y) |
| Kim J et al. | 2015 | Korea | Nov 2012 – Feb 2014 (1y 4m) |
| Farrar et al. | 2009 | England | March 2002 – June 2006 (4y 4m) |
| Martinussen et al. | 2009 | Norway | 1999 – 2005 (7y) |
| Jung et al. | 2018 | Korea | Jan 2012 – Dec 2014 (2y) |
| Dismuke et al. | 2002 | Portugal | 1992 – 1994 (3y) |
| Shmueli et al. | 2002 | Israel | July 1988 – June 1991  (3y) |
| Yin et al. | 2013 | Norway | 2000 – 2007 (7y) |
| Gaughan et al. | 2019 | England | April 2006 – March 2015 (9y) |
| Krabbe-Alkemade et al. | 2017 | Netherlands | 2006 – 2008 (3y) |
| Sutherland et al. | 2016 | Canada | April 2003 – March 2013 (10y) |
| Januleviciute et al. | 2016 | Norway | 2003 – 2007 (4y) |
| Verzulli et al. | 2017 | Italy | 2005 – 2010 (6y) |
| Hamada et al. | 2012 | Japan | April 2001 – Sept 2009 (8y 6m) |
| Cooper et al. | 2011 | England | 2002 – 2008 (7y) |
| Perelman et al. | 2007 | Belgium | 1991 – 1998 (8y) |
| Zeng | 2019 | China | Dec 2011 – July 2018 (6y 6m) |
| Theurl | 2007 | Austria | 1989 – 2003 (14y) |

**Appendix 4: Analytical Methodology - Summary by study**
**Shmueli et al. (2002):**Examined the short-term effects of ABF in the first year post implementation, across hospital volume of activity, LOS, quality of care (60-day readmission rate, in-hospital mortality 60 days and 365 days post discharge) and hospital’s real income in Israel. The effects were measured across five selected procedures: cholecystectomy, hysterectomy, hip replacement, operations on lens and heart surgeries, over a 3 year period (July 1988 – June 1991). Adopting an interrupted time series approach, they compared the effects two years before implementation (1988 – 1990) with effects one year after implementation (1990 – 1991). They estimated an OLS regression corrected for heteroscedasticity, controlling for patient characteristics (age, sex, operation type) and hospital fixed effects, with the dependent variable in logarithm form. Additionally, they estimated a logit regression model to examine the effects of ABF on readmission and mortality rates, controlling for the same patient and hospital characteristics.

**Dismuke et al. (2002):**Assess the effect of ABF payment on hospital quality, for a single DRG (Specific Cerebrovascular Disorders Except Transient Ischemic Attack) in Portugal. Using in-hospital mortality (30 days) as a quality indicator, they estimate a count data model at the hospital level using data over a 3 year period from 1992 to 1993. A Poisson model was estimated, controlling for the following hospital and patient characteristics: patient mean age, gender, case-mix, excess LOS (as measure of patient stability) hospital size, hospital volume, hospital teaching status, technological measures (CT use, ICU use), hospital status (central, district, level one), DRG payment proportion. Additionally, to overcome issues of equi-dispersion and over-dispersion, a Negative Binomial (NegBinII) regression model was estimated, controlling for the same characteristics, and the results between both models are compared.

**Theurl et al. (2007):**Assessed the impact of ABF implementation on the average hospital LOS across 20 DRGs in hospitals across nine regions in Austria. Using data over a period of 14 years from 1989 to 2003, they adopt an interrupted time series analytical approach, with model specifications consisting of hospital sector demand and supply characteristics. First, they estimate the impacts on LOS using the full data sample (pooled over regions and DRGs) including fixed regional and DRG-specific effects, and controlling for hospital characteristics (hospital cost, capacity, share of older inpatients, pharmaceutical expenditure) and fixed effects capturing time-invariant determinants of LOS (e.g. hospital accessibility in terms of distance, hospital teaching status). They assume variation in hospital technological progress over time across DRGs only, and account for DRG-specific time trends. Second, they estimate the effects of ABF on the average LOS across the 20 DRGs. They estimate a standard one-way error components models for each DRG, including a time trend to account for technological progress. The standard errors are corrected in both models to account for a possible heteroscedastic and auto correlated variance.

**Perelman et al. (2007):**Examined the result of ABF implementation in Belgium by analysing hospitals’ response in terms of in-patient LOS, medical expenditures and surgical expenditures. Interrupted time series analysis was conducted aver an 8 year period from 1991 to 1998, 4 years pre-intervention and 4 years post-intervention. First, the impacts of the reform on LOS were estimated using a model controlling for patient age, type of DRG, and a fixed effect for each sub-category of age, DRG and hospital. To control for other factors influencing changes in LOS not related to financial incentives, a linear time trend was included in the estimation. Second, the effect of cost sharing by the hospital (the share of per diem excess days the hospital must pay for itself) was estimated in the post-implementation period (after 1995), to separate the cost-sharing effect from the shifting from none to full financial responsibility. Third, the effects of changes in the use of medical resources (logarithm of total average expenditures) post reform were estimated, by controlling for hospital cost sharing effects post implementation. Finally, a socio-economic status measure, capturing the percentage of underprivileged patients within the hospital was included to control for differences in LOS based on patients’ socio-economic status.

**Farrar et al. (2009):**Used the DiD methodology to examine the impact of introducing an ABF hospital payment system across hospital trusts in England, across several outcomes measuring hospital volume, cost and quality of care. Their control group consisted of non-hospital trusts in England and providers in Scotland that did not implement the payment system. Their analysis was conducted between March 2002 and June 2006, using 5 *yearly* data points. As a result of the phased and partial implementation of the payment system in England and Scotland, the pre-treatment and post-treatment periods varied between 1 and 2 years across treatment and control groups. In their analysis, fixed effects were used to control for unobserved differences between the DRG characteristics (technology, specialty factors, patient demographics, case mix) and trust characteristics (management culture, teaching status, local population characteristics) which remain constant over time. To account for efficiency and specialisation differences of trusts and resulting differences in outcomes, fixed effects for each DRG and trust combination were created by interaction. Additionally, they controlled for characteristics of DRGs that determined their suitability for the new payment system. Finally, a two-part model with a log transformed dependent variable was estimated for the length of stay measure.


**Martinussen et al. (2009):**
Examined whether post ABF implementation and institutional organisation in Norway, there is evidence of ‘cream skimming’ (selective treatment of patients that demand few resources while providing high economic refunds) among surgical day-case DRGs. Their analysis employed patient data over a 7 year period from 1999 to 2005. They adopted an interrupted time series analysis approach, consisting of periods 3 years pre-treatment (1999 – 2001) and 4 years post-treatment (2002 – 2005). They captured cream skimming by estimating LOS as the key outcome measure, which represents waiting time for treatment and reflects patient severity per DRG. First, they tested for evidence of cream skimming following ABF implementation, by estimating a model with controls for patient characteristics (age and gender), hospital characteristics and time specific characteristics (accounting for LOS, reform and interaction term of both LOS and reform). Second, they estimated whether cream skimming has persisted following reorganizational reform in 2002, by estimating a model accounting for patient severity at the pre and post implementation period, and including hospital fixed effects that capture both observed and unobserved time-constant characteristics (by interacting time-specific and hospital-specific variables).

**Cooper et al. (2011):**Examined whether greater exposure to market competition prompted hospitals to improve their performance in terms of hospital quality, for patients diagnosed with acute myocardial infarction (AMI) in England. Over a 7 year period (2002 – 2008) they measured hospital quality in terms of changes in 30-day mortality rates among AMI patients. They used a DiD style estimator to test whether patient mortality in more potentially competitive markets has improved significantly faster post-reform, relative to less competitive markets. Using OLS, they estimated the effects of the policy, on the annual rate of change in mortality, from a break in the time trend after mid-2006, when the policy was adopted. They used two-part, *quarterly splines* (2002 as quarter 1), split at the end of the second quarter in 2006, controlling for hospital and GP fixed effects (where GPs serve as patients’ agents for hospital referral), controls for possibility of changing GP (due to new policy allowing patient choice of where to get treatment), and controls for hospital composition in high and low competition areas. Several alternative market structure indexes were constructed, and additional models estimated. Finally, a two-stage least squares IV model was estimated, where market structure was instrumented using variation of the straight-line distance from each GP to the nearest hospital, controlling for GP and hospital effects.

**Hamada et al. (2012)**:
Examined the effects of ABF in Japan, for patients with acute myocardial infarction across several hospital outcomes related to resource usage and healthcare quality: total inpatient medical charges, LOS, mortality rate, 30 day readmission rate. Using the DiD method, to incorporate time trend effects over a period of 9 years (2001 – 2009), they compared the effects across all outcomes, using the Fee-For-Service (FFS) payment as the control group, and the DRG-based payment as the treatment group. Effects were captured at 3 years pre-intervention and 6 years post-intervention. To account for cluster-related correlation between hospitals, they estimated a multilevel, mixed-effect, regression model for all the continuous outcomes (e.g. medical charges, LOS) and a multilevel, mixed effect, logistic model for all the binary outcomes (e.g. readmission, mortality). Both regression models accounted for individual patient level characteristics (sex, age, Charlson Comorbidity Index, received percutaneous coronary intervention or coronary artery bypass grafting) as well as hospital level characteristics in their estimation.


**Yin et al. (2013):**Examined the effect of ABF on hospital length of stay for elderly patients with ischemic heart diseases (angina pectoris, congestive heart failure, myocardial infarction) in Norway. They conducted patient level analysis over a period of 8 years from 2000 to 2007, limited to three ischemic heart diseases to reduce intra-diagnostic heterogeneity in LOS. They focused on capturing the effects a few years post the implementation period. Initially, they estimate a model with the logarithm of LOS as the dependent variable, controlling for exogenous effects, unobserved individual effects, patient characteristics (age, gender, age squared) hospital catchment areas which may influence LOS due to patient transfers, and include a linear time trend (capturing technological improvements, nursing home and outpatient care developments, patient preferences for shorter hospital stays). Additionally, they estimated a pseudo panel model, which included the IV method. The patient data was aggregated into a pseudo-panel with repeated observations over time, by grouping individuals into pseudo-cohorts based on a fixed membership over the observation period. A model with average logarithmic LOS as dependent variable was estimated, controlling for fixed cohort effects over study period, using IV estimators to account for the eliminated time-invariant variables and ignored variation across individuals, following data transformation. In their final estimated model, some of the exogenous variables (age, comorbidities, DRG weight, angina pectoris and congestive heart failure) were allowed to be correlated with some of the individual-level random effect.

**Kim H et al. (2015):**

Examined procedure level effects of ABF payment at a single hospital in Korea, for appendectomies over a 12 month period (from July 2012 to June 2013). They measured the early effects across several outcomes consisting of clinical outcomes (LOS, complication rate, outpatient visits, readmission), and the medical costs. They compared the changes across all outcomes between two groups, 6 months pre-intervention and 6 months post-intervention. Their analysis consisted of simple descriptive methods with all continuous outcomes compared using a Students t-test, and categorical outcomes compared using the Pearson chi-square test. They used the Fisher exact test if the frequency of the outcome of interest was less than 5, it was linearly associated, or it consisted of more than two categories. Analysis of frequencies and percentages were used to examine the distribution of all the categorical outcomes, and continuous outcomes were presented in terms of their mean and standard deviation.

**Kim J et al. (2015l:**

Evaluated the impact of the ABF payment system on the use of medical resources and the rate of adverse events for patients undergoing laparoscopic appendectomy in Korea. Data for patients over a 16 month period from November 2012 to February 2014 were compared and differences captured across a number of outcomes: LOS, cost of hospital stay, outpatient visits, readmission rate, adverse events, complication rate during initial hospitalisation and 30 day readmission rate. Simple descriptive analysis methods were employed. All continuous outcomes were analysed using the Students t-test, and categorical outcomes were compared using the Pearson chi-square test.
 **Allen et al. (2016):**
Used the DiD methodology to analyse the impact of introducing a Best Practice Tarriff (BPT) for a day case procedure, cholecystectomy, with a *control group* of non-incentivised procedures recommended for day case treatment. Analysis was conducted from December 2007 to March 2011, using *40 monthly data points*. The pre-intervention period consisted of 28 months, and post-intervention 12 months. To estimate average effects, they estimated a weighted least squares regression with fixed-effects, at *procedure level*. To test for the *unobserved temporary component* (a temporary change in the number of procedures prior to implementation), they interacted cholecystectomy and the anticipation period at month 25 when the policy was announced. Additionally, they performed a test for *common trends* between cholecystectomies and control procedures. This was done by testing the significance of the interaction between cholecystectomy dummy and a linear trend measuring the number of months since the beginning of the data period, to the month before the policy was announced, separately for each dependent variable. Additionally, a spline regression was applied to test for changes in the outcome trend over time, by creating knots at month 25 and month 29, to capture the anticipation and BPTs effects, respectively.

**Januleviciute et al. (2016):**Examined the choice of same-day discharge care versus overnight stay across hospitals under the ABF payment system in Norway, where prices were differentiated by admission type. They estimated several fixed-effect models (logarithmic form) to examine the changes in the number of patients treated within the DRGs over a 5 year period from 2003 to 2007, with year 2003 as the reference year. In their analysis, they controlled for unobservable heterogeneity across hospitals and DRGs, by including hospital and DRG fixed effects. Additionally, a linear time trend interaction with each DRG was estimated, to control for changes in volume across DRGs over time, to separate the price change effect on activity volume from other changes over time.

**Sutherland et al. (2016):**Examined the changes following the implementation of ABF in Canada (British Colombia) across a number of hospital outcomes: volume of care (sum of weighted patient cases), efficiency of care (total LOS, alternative level of care days), quality of care (readmission, in-hospital morality). Their analysis was conducted over a period of 5 years, consisting of 65 financial time periods (months), consisting of 26 pre-implementation and 39 post-implementation periods. They used interrupted time series analysis of segmented regression models, accounting for correlation within both Health authority (geographically-defined regions) and hospital over time. Hospital-level multivariate linear regression model was used to measure hospital volume, controlling for time of policy implementation, interaction representing change over time concurrent to policy implementation and fixed hospital and health system effects.
Similarly, multivariate linear regression was used to measure efficiency, controlling for patient case-mix, hospital fixed effects and health system effects, assuming patients were nested within their fixed hospital in a fixed Health Authority. Finally, to capture the effects of quality, individual-level multivariate logistic regression models were estimated with controls for patient, hospital and health system effects. To assess policy impact differences across regions, sensitivity analysis was performed.

**Verzulli et al. (2017):**Examined the impact of a policy change in the Italian region Emilia Romagna, following a price increase across several DRGs, and its impact on public hospital performance across several outcome measures: volume of activity, waiting times (waiting more than 6 months for treatment), average LOS. They examined a one-time increase in DRG-prices, using 6 years of data (2005 to 2010) for all acute patients who received elective inpatient treatment. To capture the differences in effects of the price change across the DRGs, 164 DRGs (medical and surgical) which were affected by the price change, were compared to 115 DRGs (medical and surgical) that were unaffected by the price change. They employed a difference-in-differences approach, with outcomes expressed in logarithmic form, controlling for DRG-by-hospital fixed effects, time-invariant DRG characteristics across each hospital, year fixed effects, with baseline year set to 2006, and controls for common DRG shocks in all hospitals. The key explanatory variables were used to capture price changes in the short and medium-run by interacting the price change in the short-run over the first 2 years after the policy (2007 and 2008), and the medium-run price change interacted in the two subsequent years (2009 and 2010). Additionally, to control for possible time-varying hospital characteristics common to all DRGs (e.g. employed staff), and other unobserved DRG characteristics; hospitals and year fixed effects and a linear time trend for each DRG were also interacted. Additional estimation was conducted, to capture differential short and medium post-policy effects across hospitals with differing administrative and financial autonomy of hospitals, and effects also estimated for hospitals based on their level of bed occupancy 1 year before the policy was implemented.

**Krabbe-Alkemade et al. (2017):**Used the DiD approach to examine the impact following the implementation of ABF payment system (market competition) across hospitals in the Netherlands. Their analysis was conducted over a 3 year period from 2006 to 2008. Their pre-treatment period consisted of 2 years, and post-treatment period of 1 year. Their treatment group consisted of DRGs that were transferred from the old payment system i.e. the budget-based system, and the control group consisted of DRGs that remained in the budget-based system. To avoid the impacts of price differences on cost information, price levels from 2005 (implementation year) were applied across all 3 years. They examined the impact of market competition across four key hospital level outcomes: volume, number of cases, total costs and average costs, controlling for hospital fixed effects. They also controlled for the clustering of DRGs on hospital level by adjusting the standard errors. Additionally, hospital characteristics were included in estimation, to examine hospital heterogeneity.

**Jung et al. (2018):**Examined the effects following the introduction of ABF payments across a number of procedures (caesarean sections, hysterectomies, adnexectomies) in the field of obstetrics and gynaecology in Korea. They looked at the resulting changes across several key outcomes: total number of operations, number of emergency operations, LOS, spill over rate, readmission rate and number of simultaneous operations.
They compared the changes across all outcomes over 24 months from 2012 to 2014, with 18 month pre-implementation and 6 month post-implementation periods. They used simple descriptive analytical methods. Analysis of frequencies and percentages were used to examine the distribution of each categorical outcomes, and chi-squared test was performed to examine the association between the outcomes and the DRG-payment system. For comparison of average values and standard deviations across continuous outcomes, the Student’s t-tests were performed.

**Gaughan et al. (2019):**Used DiD and synthetic control methods to examine the impact of the introduction of same-day discharge price incentive across 32 incentivised conditions in England, with a *control group* of non-incentivised conditions with similar characteristics. They conducted their analysis between 2006 and 2014 using 36 data points consisting of *hospital quarters*. The pre-intervention period consisted of 14 (data for 2 hospital quarters prior implementation were excluded), and post-intervention 20 hospital quarters.
For each incentivised condition, DiD and synthetic control models were estimated. For DiD estimation the conditions selected as control, must satisfy the parallel trends assumption. The DiD estimation model accounts for condition-specific seasonal effects (spring, summer, autumn, winter) and hospital fixed effects (management quality, local demand). All models capture the *average treatment effects* and are estimated as linear probability models with standard errors clustered at hospital level. Additionally, *synthetic control models* are estimated, where each control condition is treated as if it were incentivised and pooled with the actual incentivised condition. The root mean squared prediction error was calculated at pre and post intervention periods, and p-values constructed to allow for comparison of estimates between the different DiD and synthetic control model estimates.

**Zeng et al. (2019):**Evaluated the effects of ABF-based payment in a pilot hospital in Beijing, China, across 107 DRGs, to determine whether the payment reform resulted in a profit or loss for the hospital. The effects were evaluated at *patient level*, over a period of 6 years and 6 months, from December 2011 to July 2018. Descriptive methods (mean, median) were used to present the patient demographics, LOS, average inpatient expenditure, average DRG unit price, and the average patient self-pay expenditure (medical expenditure covered by patients and not health insurance). All the DRGs were compared in terms of being profit or loss making to the hospital. Finally, a logistic regression analysis was estimated to determine whether the hospital was making a profit or a loss following the payment reform, and identified which of the key factors (average inpatient expenditure, average LOS, drug expenditure, expenditure of medical consumables, years of pilot implementation, average DRG unit price, average self-pay expenditure, age and gender) impacted most on the profit and loss situation.
